# Development of a complex Interdisciplinary Nurse-coordinated SELf-MAnagement (INSELMA) intervention for patients with inflammatory arthritis

**DOI:** 10.1186/s12913-023-10463-1

**Published:** 2024-01-17

**Authors:** Jette Primdahl, Ann Bremander, Oliver Hendricks, Mikkel Østergaard, Kristine Marie Latocha, Lena Andersen, Kim Vilbaek Jensen, Bente Appel Esbensen

**Affiliations:** 1 Danish Hospital for Rheumatic Diseases, University Hospital of Southern Denmark, Engelshøjgade 9A, Sønderborg, 6400 Denmark; 2 Department of Regional Health Research, University of Southern Denmark, Odense, Denmark; 3 Sygehus Sønderjylland, University Hospital of Southern Denmark, Aabenraa, Denmark; 4 Spenshult Research and Development Centre, Halmstad, Sweden; 5 Section of Rheumatology, Department of Clinical Sciences Lund, Lund University, Lund, Sweden; 6 Copenhagen Center for Arthritis Research (COPECARE), Center for Rheumatology and Spine Diseases, Copenhagen University Hospital - Rigshospitalet, Glostrup, Denmark; 7 Department of Clinical Medicine, Faculty of Health and Medical Sciences, University of Copenhagen, Copenhagen, Denmark; 8 Patient Research Partner, Sønderborg/Glostrup, Denmark

**Keywords:** Rheumatoid arthritis, Psoriatic arthritis, Spondyloarthritis, Coherence, Multi-disciplinary, Goal setting, Patient-specific functional scale, Nonpharmacological, Self-efficacy

## Abstract

**Background:**

Apart from a consistent focus on treating inflammation, patients with inflammatory arthritis (IA) report a range of unmet needs. Many experience not only residual symptoms but also various other physical, psychological, and social effects. Therefore, this study aimed to develop a complex Interdisciplinary Nurse-coordinated self-management (INSELMA) intervention for patients with IA, as an add-on treatment to usual outpatient care for those with substantial disease impact.

**Methods:**

This study followed the British Medical Research Council’s updated framework for developing complex interventions. The process encompassed the following steps: (1) The evidence base was identified; (2) workshops were held, involving 38 relevant stakeholders (managers, physicians, nurses, physiotherapists, occupational therapists, social workers, psychologists from hospitals and municipalities, and two patient research partners), to discuss and further develop the preliminary ideas; (3) relevant theories were identified (i.e., self-efficacy, acceptance and commitment therapy, and health literacy); (4) the intervention was modeled and remodeled and (5) the results, describing the final INSELMA intervention and outcomes.

**Results:**

The INSELMA intervention encompasses an initial biopsychosocial assessment, which is performed by a rheumatology nurse. Then, activities that the participant wishes to improve are identified and goals are set. The nurse refers the participant to a multidisciplinary team and coordinates their support and relevant services in the participant’s municipality. In addition, the health professionals have the opportunity to hold two interdisciplinary conferences during the intervention period. The participant and the health professionals work to achieve the set goals during a 6-month period, which ends with a status assessment and a discussion of further needs. The INSELMA intervention aims to increase self-management, reduce the impact of IA (e.g., pain, fatigue, sleep problems, and absenteeism), and increase self-efficacy, quality of life, mental well-being, work ability, and physical activity.

**Conclusions:**

The development of the INSELMA intervention involved stakeholders from two Danish rheumatology outpatient clinics, patient research partners and municipalities. We believe that we have identified important mechanisms to increase the self-management and quality of life of people with IA and to decrease the disease impact in those who are substantially affected. The health professionals involved have developed competences in delivering the intervention and it is ready to be tested in a feasibility study.

**Supplementary Information:**

The online version contains supplementary material available at 10.1186/s12913-023-10463-1.

## Background

This study focused on the development of a novel self-management intervention for people with rheumatoid arthritis (RA), psoriatic arthritis (PsA) or axial spondylarthritis (axSpA), all of which are forms of inflammatory arthritis (IA). They share certain characteristics, such as swelling, joint tenderness and stiffness, and reduced mobility [1–3]. In Denmark, approximately 0.6% of the population are diagnosed with RA [4], while 0.2% have PsA [5], and 0.4–1.5% have axSpA [6, 7].

Despite major improvements in early diagnosis, the initiation of antirheumatic pharmacological treatment, and a consistent focus on treating inflammation [8], people with IA still have unmet needs. Approximately 30% do not respond sufficiently to or tolerate treatment with antirheumatic drugs, and thus, they do not achieve remission or a state of low disease activity [9–12]. Pain, fatigue, sleep problems, anxiety, functional disability, and reduced participation in social activities and paid work are commonly experienced problems. Moreover, even patients considered to be in a state of remission or low disease activity often experience substantial physical, psychological, and social impact of IA in their everyday lives [2, 3, 12–14]. In addition, people with IA have an increased risk of extra-articular manifestations and comorbidities, such as cardiovascular disease, diabetes, osteoporosis, infections, chronic widespread pain, peptic ulcers, depression, and certain malignancies [15–20]. Furthermore, IA often imposes a substantial socioeconomic burden caused by expensive medication, decreased social participation, and reduced ability to work [21–24].

Notably, people who experience substantial impacts from arthritis require support to self-manage their condition and increase their quality of life [25–28]. In 2020, the European Alliance of Associations for Rheumatology (EULAR) published updated recommendations for the role of nurses in the management of IA [29]. One recommendation is that the patient should have access to nurse-facilitated needs-based education to improve their knowledge of their arthritis and its management throughout the disease course. In addition, nurses should support patients’ self-management skills, to increase their self-efficacy [29]. In general, self-management is associated with patient activation, a person-centered approach, and shared decision making [27]. In this study, a person-centered approach is inspired by McCormack et al., that the health professionals (HPs) work with patients to identify their beliefs and values, demonstrate engagement and empathy and work to involve the patients in shared decision making and provide holistic care [30]. For a patient to manage their disease and well-being as effectively as possible, they require not only information about the disease but also support in managing the treatment, lifestyle changes, and the potential physical, emotional, and social impacts of their chronic condition [27, 31, 32]. Self-management support encompasses biopsychosocial assessment, goal setting, and action planning, which are also central elements in the rehabilitation process [27, 33].

Because the challenges faced by people with IA can be multi-faceted, they require support from multiple professionals, such as rheumatologists, nurses, physiotherapists (PTs), occupational therapists (OTs), social workers, and psychologists [28, 34]. The management of IA requires coordination and information between the patient, their relatives, and various HPs in primary and secondary care [27, 35]. However, patients often experience a lack of coherence across both specialties and primary and secondary care related to insufficient communication and coordination between the HPs involved [36–39].

Furthermore, evidence is lacking regarding the effect of self-management interventions that target patients with IA who experience substantial impact from their condition. There is also a lack of research on outpatient self-management interventions for people with IA that involve multiple HPs. Thus, there is a need to develop and test coordinated interdisciplinary self-management interventions that target people with IA who experience substantial impact from their arthritis. The Medical Research Council (MRC) has published a useful framework for developing and evaluating such complex interventions involving multiple components and multiple professionals [40].

Therefore, the objective of this study was to develop an interdisciplinary nurse-coordinated complex self-management intervention as an add-on to usual outpatient care for patients with IA who face substantial impacts. The overarching focus of the study was to support these patients’ self-management ability and reduce the impact of their arthritis.

## Methods

### Setting

This study involved two rheumatology outpatient clinics in Denmark, namely the Danish Hospital for Rheumatic Diseases, Sønderborg and the Center for Rheumatology and Spine Diseases at Copenhagen University Hospital, Rigshospitalet - Glostrup. The research group comprised researchers with a range of professional backgrounds (two rheumatologists, a PT, and three nurses) and two patient research partners (PRPs) with IA (a man and a woman, one from each hospital). The two PRPs both have had RA for many years; they have experienced a substantial impact of arthritis and are not able to work. Both have previously participated as PRPs in several studies and are involved in the Danish Rheumatism Association. Thus, they have contact with many other people who have various types of IA.

The Danish healthcare system operates across three administrative levels: (1) the state, comprising the regulatory and supervisory body; (2) five regions, responsible for in- and outpatient hospital care (secondary health care); and (3) 98 municipalities, responsible for public health, prevention, general rehabilitation, home nursing care and social services (primary care). Citizens can see free of charge their general practitioners (GP). GPs work in primary care, but are reimbursed by the Regions. For patients with IA, standard outpatient care typically involves scheduled face-to-face or telephone consultations with a rheumatologist or a rheumatology outpatient nurse once or twice a year. Additional consultations are available for patients who experience flare-ups or have medication-related concerns. Rheumatologists primarily focus on diagnosis and pharmacological treatment. The nurses perform joint assessments, evaluate blood samples, and focus on adherence and side effects of the pharmacological treatment. However, they have limited time to address psycho-social issues, such as pain and fatigue management. In cases where patients require supervision by a PT or OT, the patient is referred to primary care.

### Study design

We planned the development process in accordance with the MRC’s updated framework for developing and evaluating complex interventions [40], as the intervention would involve multiple interacting components and various HPs. The development and evaluation of the complex intervention in the overall study consisted of the following four phases: the development or identification of the intervention, feasibility, evaluation, and implementation.

This article reports on phase one; the development of the intervention, which consisted of the following five steps [40]: (1) identifying the evidence base; (2) holding workshops involving relevant stakeholders; (3) identifying relevant theories; (4) modeling and remodeling the intervention; and (5) reporting the results (describing the final intervention and outcomes to be tested in a subsequent feasibility study). In the following subsections, each step is described in accordance with published criteria for reporting the development of complex interventions [41].

### The development of the intervention

#### Identifying the evidence base

We did not find existing evidence in available databases of a relevant self-management intervention for patients with IA and substantial impacts from their disease. We therefore performed a comprehensive scoping review of the literature in the Medline, CINAHL, Embase, PsycINFO, and Cochrane Library databases, to guide the development of a novel intervention [40].

A research librarian from the University of Southern Denmark supervised the literature search. A protocol for the scoping review was published in the Open Registries Network (OSF Registries) [42]. The following two research questions guided our systematic literature search for the scoping review: (1) What are the patients’ perspectives on their self-management support needs for living with IA and (2) What content is included in self-management interventions targeting people with IA (theory/theoretical approach, mode of delivery, duration and frequency)? Respectively, a total of 31 and 33 articles were included regarding research questions 1 and 2. The details of the scoping review are reported separately [43]. The overall results of the scoping review are presented in Table 1.

**Table 1:** Summary of the results of the scoping review [43]

Self-management support needs in people with inflammatory arthritis	Content of self-management interventions for people with inflammatory arthritis
Patients require self-management support regarding the impact of the disease as well as pharmacological and nonpharmacological treatment, support from family and friends, and support regarding paid work-related issues. Patients value continuity in their care to establish a positive relationship. Patients have asked for different modes of support (i.e., face-to-face in one-to-one or group sessions, individual or group-based online meetings, or mail or phone support).	Self-management interventions have been described as patient-centered. Self-management interventions have been based on a variety of theories related to self-management, such as self-efficacy, self-care behavior, cognitive behavioral therapy, cognitive restructuring techniques, the health belief model, social learning theory, social cognition theory, and behavioral change theory. Self-management interventions should be problem-focused as well as goal- and action-oriented.

Based on a previous study on barriers to and facilitators of coherence in rehabilitation [39], we planned to train experienced rheumatology nurses as coordinators in the INSELMA intervention, to ensure continuity and coherence across the different professionals involved as well as across primary and secondary health care. We also planned for the intervention to start with an initial assessment, goal-setting, and action planning [28, 33].

### Workshops involving relevant stakeholders

In the fall of 2021, we planned and conducted workshops involving key stakeholders. The aims were to discuss and further develop initial ideas for a relevant and feasible intervention, with an awareness of the local context, to create ownership, and to ensure the development of a feasible intervention. The workshop participants helped to define the content and suggested how to outline the intervention. They also discussed relevant outcome measures and the need to develop the competence of the HPs who would deliver the intervention. Initially, we planned two extensive workshops, one at each hospital. Due to COVID-19 restrictions we were not allowed to mix people from various settings. We thus ultimately held six smaller workshops, four of which were face-to-face with HPs (two workshops at each hospital) and two online (with a patient representative and a PRP and HPs from various municipalities). In total, 38 professionals, a patient representative and a PRP participated. Table 2 presents an overview of the participants.

**Table 2:** Overview of the participants of the six workshops

Background	Gender	Age	Experience within rheumatology
Rheumatology nurses (n = 17) Physicians and rheumatologists (n = 10) PTs (n = 5) OTs (n = 3) Social worker (n = 1) Manager of municipal rehabilitation center (nurse) (n = 1) Psychologist (n = 1)	Female (n = 27) Male (n = 11)	28–65 years (median 52.4)	6.5–31 years (median 18)
Patient representatives *(n = 2)	Female (n = 1) Male (n = 1)	In their 50 and 60 s; both had rheumatoid arthritis	Disease duration: more than 30 years

*One of the patient representatives is the male patient research partner who participated in the project group. PT: Physiotherapist; OT: Occupational therapist. Two of the physiotherapists, a physician, and a nurse working as manager of a municipal rehabilitation center, all worked in different municipalities and the psychologist worked at a rheumatology rehabilitation center

The face-to-face workshops consisted of presentations of how to support self-management, initial ideas for the intervention, creative exercises with sticky notes, guided reflections, and dialogue. The initial ideas encompassed the initial assessment and goal setting, the opportunity for self-management support from PTs and OTs, opportunities for team conferences, and coordination and support by an experienced rheumatology nurse. Each workshop was facilitated by a moderator, namely first author, Professor Jette Primdahl (JP) or last author, Professor Bente Appel Esbensen (BAE). The online workshops consisted of presentations, guided reflections, and discussions. The dialogue from the workshops was audio-recorded and the sticky notes were transcribed. In the software program NVivo version 10 (QSR international), the transcribed text was coded into the following predefined themes and summarized: (1) assessment of patients’ needs and resources; (2) content of the intervention and outcomes; and (3) communication with primary care.

#### Highlights of the workshops

The workshop participants emphasized that the intervention should target patients who have been diagnosed for at least 2 years, and where pharmacological treatment is expected to be stable. Furthermore, they recognized that patients who suffer substantial impacts from their IA or struggle with acceptance of life with arthritis, require self-management support to manage the various symptoms and limitations.

The hospital staff involved discussed whether an initial holistic biopsychosocial assessment, goal setting, and action planning should be performed by each of the professionals involved or by the coordinating nurse, followed by appropriate referrals to a PT, OT, social worker, or rheumatologist. They highlighted that a person-centered approach was crucial and valued the idea of a rheumatology nurse to assist the patient in coordinating support from both various professionals and across primary and secondary health care. In addition, they believed that the coordinating nurse could support each patient toward goal achievement and ensure continuity in their care, which is supported by the literature [44, 45],

Furthermore, some of the participants in the workshops (HPs, social worker and physicians) mentioned that patients with IA might reach a state where they feel emotionally “worn out” after attempting to manage everyday life with IA and navigate the health and social care systems for some time. Thus, they pointed to the need for HPs to be able to address the psychosocial challenges, namely through a cognitive behavioral approach, in alignment with the EULAR recommendations [28]. In addition, some of the HP participants suggested that self-efficacy could be used as the learning theory [46] and that it could also be an outcome. Other suggested outcomes were measures of pain, fatigue, and quality of life. Later in this manuscript, under the heading “A subsequent feasibility study”, we describe how to identify the INSELMA target group.

Discussions in the workshops also addressed how to signpost and communicate with colleagues in the municipalities and how the HPs could keep up-to-date with the services available in each patient’s municipality, given that each hospital collaborates with several municipalities. The discussions revealed a need for increased coordination and communication across primary and secondary health care.

Moreover, the HP participants described a need for increased competencies among the HPs at the hospitals who were to deliver the intervention. These competencies included: the ability to support self-management, self-efficacy, health literacy, and symptom management, a cognitive behavioral approach, knowledge about when to refer to other HPs, and knowledge of social support opportunities.

#### Identification of relevant theories

In accordance with the literature review and input from the workshops, we wanted the initial biopsychosocial assessment to be person-oriented and for the goals agreed upon between the HPs and the participant to be based on the participant’s needs, values, and preferences [33, 47]. A cognitive behavioral approach was mentioned as relevant. We chose acceptance and commitment therapy (ACT) [48, 49], as it focuses on psychological flexibility. We considered an ACT approach to be relevant rather than a traditional cognitive behavioral therapeutic approach, as patients with IA should work toward accepting some degree of impact of their arthritis on their everyday life. Evidence suggests that ACT is helpful in both improving self-management and lifestyle in patients with chronic diseases and in managing pain, anxiety, and depression, for example [48, 50–55]. In addition to the concept of self-management [25, 32] and ACT, we chose the theory of self-efficacy [46], in accordance with findings from the scoping review [43] and workshops. We also included the concept of health literacy [56], to guide the content in the intervention and explain the anticipated effects. Participants’ health literacy level was expected to affect their ability to be actively involved in the management of their condition and health [57]. The identified and selected theoretical and conceptual approaches are described in Table 3.

**Table 3:** Theoretical approaches selected to support the intervention

Theoretical and conceptual approaches	Short description
**Self-management**	Self-management focuses on patients’ active involvement in their own health and care. The idea is to support people with IA to be able to manage the symptoms and impacts of IA on their everyday life and to maintain their independence and quality of life [25, 32]. Professor and health psychologist Julie Barlow and her colleagues defined self-management as “the individual’s ability to manage the symptoms, treatment, physical and psychosocial consequences, and lifestyle changes inherent in living with a chronic condition. Efficacious self-management encompasses the ability to monitor one’s own condition and to affect the cognitive, behavioral, and emotional responses necessary to maintain a satisfactory quality of life. Thus, a dynamic and continuous process of self-regulation is established” [25]. The focus on self-management is linked to a person-centered approach where the HPs work with patients’ beliefs and values, show engagement, have an empathic presence, work toward shared decision making and to provide holistic (biopsychosocial) care [30] The HPs’ behavior and attitude can become a barrier to the patient’s self-management ability due to, for example, blame, guilt, and excessively high demands.
**Self-efficacy**	Self-efficacy refers to a person’s belief or confidence in his or her capacity to solve a specific problem or to perform a behavior necessary for attaining a specific outcome [45]. A person’s self-efficacy is thought to affect all types of experiences, including the goals they strive for and the amount of energy spent toward achieving a goal; furthermore, self-efficacy beliefs affect the likelihood of reaching a specific level of behavior. Self-efficacy beliefs are also thought to vary, depending on the specific problem or behavior, and to be influenced by the social context. A person’s self-efficacy belief can be affected in the following four ways: one’s own experience of performance accomplishment; role modeling (vicarious experience), which refers to seeing others perform a specific behavior or solve a problem; verbal support or verbal persuasion; and encouraging a person or emotional and physiological feedback (e.g., how one feels when doing physical exercise) [46].
**Acceptance and commitment therapy (ACT)**	ACT focuses on living a meaningful life despite fluctuating symptoms, such as pain and fatigue. ACT involves the following six processes: (1) acceptance (experiencing what is happening without the need to try to control or avoid unpleasant feelings and thoughts); (2) cognitive diffusion (detaching from thoughts and separating from behavior, avoiding seeing thoughts as “true” and thus directing one’s behavior); (3) awareness of the present moment (being aware of what is happening internally and externally– thoughts, feelings, and sensations, and also what is happening around you); (4) self as context (attempting to take an observer perspective on unhelpful thoughts and limiting ideas); (5) values (becoming aware of what is important and meaningful in one’s own life); and (6) committed action (letting values and goals direct one’s actions, even if unpleasant thoughts and feelings occur) [48, 49, 55].
**Health literacy**	Health literacy is defined as *“the combination of personal competencies and situational resources needed for people to access, understand, appraise and use information and services to make decisions about health”* [58]. The type of health literacy can be described as (1) functional (to possess literacy, knowledge, and other skills sufficient for acquiring and acting on health-related information and the recommended use of health care services); (2) interactive (the skills required to extract, understand, and discriminate health information from different sources and apply the information to changing circumstances); and (3) critical (advanced cognitive and social skills that can be applied to critically analyze health information from different sources and to use it to gain control over personal health decisions and their consequences) [58].

HPs: health professionals

#### Modeling and remodeling the intervention

According to the framework for the development and evaluation of complex interventions, a program theory is required to describe how an intervention is expected to lead to its anticipated effects and under what conditions [40]. Accordingly, and based on the results of the evidence base, input from workshops, and relevant theories, we developed a program theory and a detailed manual.

### Development of our program theory

The program theory must describe the resources, key components of the intervention, how elements in the context are expected to influence the mechanisms in the intervention, and the conditions under which these mechanisms might influence the context [40].

**Fig. 1:**
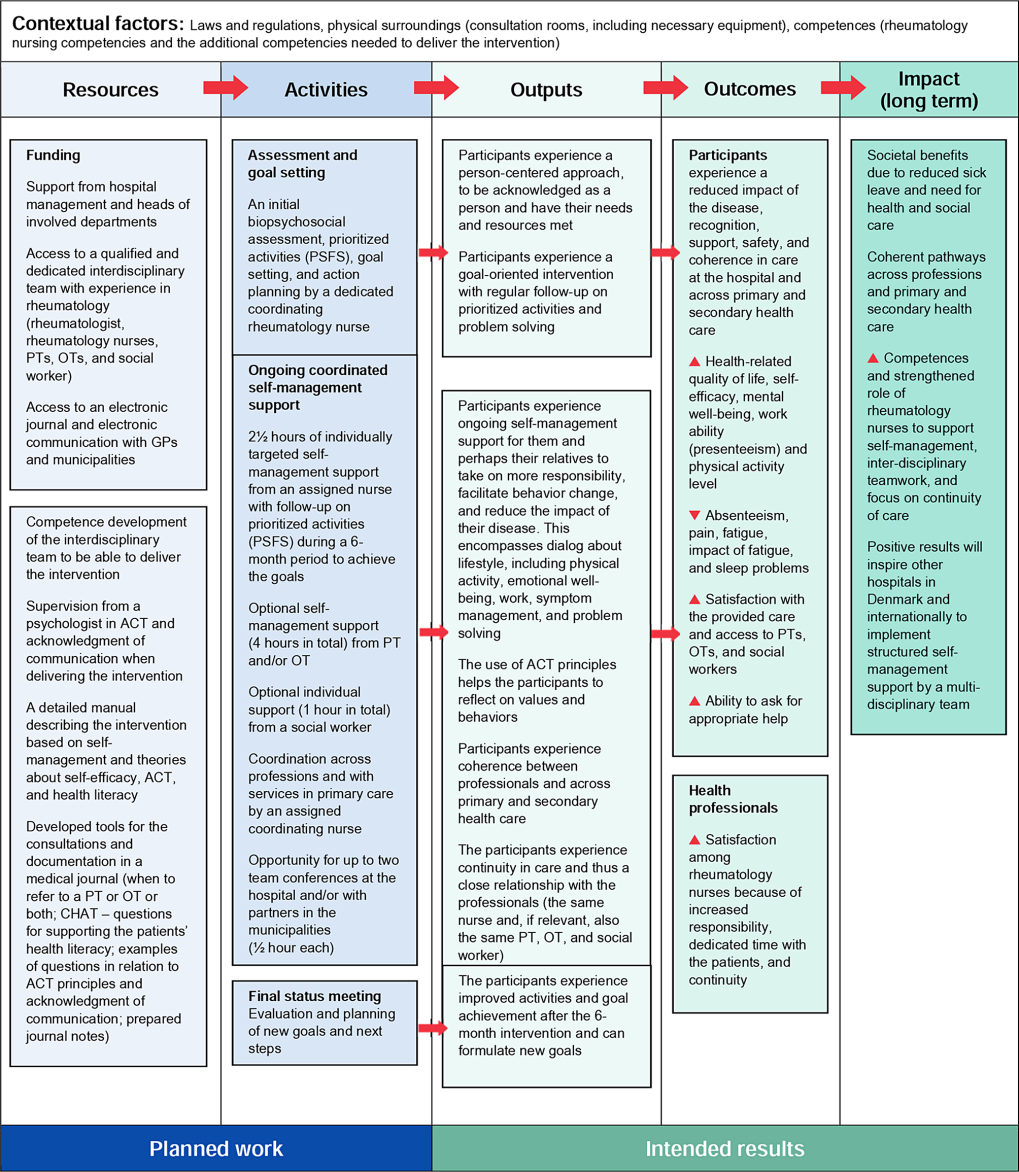
Basic logic model of the intervention [46] PT: physiotherapist; OT: occupational therapist; GP: general practitioner; ACT: acceptance and commitment therapy; CHAT: Conversational Health Literacy Assessment Tool; PSFS: Patient-Specific Functional Scale. Resources refer to the available human, financial, organizational, and community resources. Activities refer to the processes, tools, events, technologies, and actions in the intervention for bringing about the intended changes and results. The intended results are described as following: Outputs, which refer to what one aims for in the intervention; they are the specific changes in the participants’ behavior, knowledge, skills, status, and level of functioning. Outcomes refer to specific outcome measures and expectations as to whether they will increase or decrease, based on the described resources and activities. Impacts are the intended or unintended changes in organizations, communities, or systems as a result of the intervention in the longer term (i.e., 7–10 years)

The program theory can be described in a logic model, as was described by the W.K. Kellogg foundation [59]. A logic model is a visual way of presenting how an intervention works, the relationships between the required resources, and the expected outcomes and impacts. The authors JP, Kristine Marie Latocha (KML) and BAEdrafted the basic logic model and the other authors commented on it. The defined goals will be supported by identifying up to five activities during the initial consultation that the participant wishes to improve or change. The Patient Specific Functional Scale (PSFS) [60] will be utilized to discuss and assess the performance of these defined activities during face-to-face nursing consultations. The tool will aid in maintaining a continued focus to enhance performance of the activities and work towards achieving the mutually agreed-upon goals. Self-efficacy is considered to mediate self-management [31]. The HPs tried to use own experiences, role models, verbal persuasion and dialogue about the participants’ physical, emotional and physiological reactions to behaviors or situations. We hypothesized that the HPs’ use of ACT principles in their communication could help the participants to become aware of their values, how they had managed their symptoms and roles in everyday life, what they had avoided so far and how to commit to future actions. We expected the participants’ health literacy to affect their ability to increase their self-efficacy beliefs and enhance their self-management of symptoms and ability to live with a chronic disease, thus improving their quality of life [57]. The coordinating nurses could utilize questions from the Conversational Health Literacy Assessment Tool (CHAT) [61] to identify the participants’ health literacy levels and potential challenges, pinpointing areas where support would be needed. The program theory is described in Fig. 1.

### Development of a manual

The development of a comprehensive, detailed manual describing the intervention was achieved through an iterative process with input on several occations from the research team, including the two PRPs, the involved HPs, and an international advisory board. The project group held the ultimate responsibility and made the final decisions on the content of the final intervention. The manual was reviewed and revised several times during this process. The description of the intervention in the manual follows the template for the Intervention Description and Replication (TIDieR) checklist and guide [62], supplemented by the PRECIS-2 tool [63]. Furthermore, the required competence development of the nurses, PTs, and OTs who are to deliver the intervention is described in the manual. We finalized the manual in December 2021.

### Patient and public involvement

Two PRPs participated in the overall project group, as recommended by EULAR [64]. They were also involved in planning the study and developing the intervention, where they also participated in one of the workshops. They have provided significant input in terms of the content and feasibility of the intervention and commented on draft versions of the article. In addition, another patient representative was involved together with one of the PRPs as crucial stakeholders in an online workshop, where they provided input regarding the content and outline of the intervention. The idea for the study and the intervention received positive feedback from users in the User council at the research department at the Danish Hospital for Rheumatic Diseases.

## Results

The final INSELMA intervention consists of three parts, which are to be delivered over a 6-month period, as described in Table 4. The following tools to support the fidelity of delivering the intervention were developed: questions for a biopsychosocial assessment, open questions for exploring the patients’ values and stimulating reflections, based on recognizing communication and ACT principles, the CHAT tool [61] to address health literacy, a chart developed by the involved PTs and OTs to help identify when it is relevant for nurses to refer the participant to a PT or OT, and material about social support opportunities developed by the social worker who participated in the workshops. Baseline information and outcomes two weeks after the close-out consultation were defined based on the logic model. The outcomes encompass physical disability, physical activity, lifestyle, impact of the disease, pain, fatigue, self-efficacy, mental well-being, health-related quality of life and work ability. The selected outcomes and specific outcome measures are described in Supplementary Table 1 [65–80].

**Table 4:** The three parts of the INSELMA intervention

Part of the INSELMA intervention	Aim	Content
**Initial consultation** (1.5 h)	To perform a biopsychosocial assessment, define activities, and agree on goals and initial action planning in a person-centered approach, where the participant experiences being acknowledged and having their needs and resources being met	A registered nurse with rheumatology experience is assigned to each patient to ensure continuity. Together with the patient and potentially also their relatives, the nurse performs an initial biopsychosocial assessment and with the patient, defines up to five activities that the patient would like to improve based on the PSFS (60). A subsequent shared goal-setting process is followed by action planning. The nurse informs the participant about the opportunities for individual support from the nurse, a PT, OT, or social worker at the hospital to achieve the patient’s goals. The nurse can also contact the participant’s rheumatologist, if needed. In addition, the nurse helps identifying other opportunities for supporting the achievement of the goals they have agreed upon in his/her municipality.
**Individually adapted continuous support over the following 6 months** (2.5 h for the nurse, 4 h from the PT and/or OT, and 1 h from the social worker)	To ensure coherence and goal achievement through individually targeted self-management support by an assigned rheumatology nurse, problem solving and coordination across rheumatology professionals, and also across primary and secondary health care	The coordinating nurse provides continuous individual education and self-management support to the participant and his/her relatives, to help solve problems and achieve the goals agreed upon. In the dialogue, the nurse can use examples of questions that address biopsychosocial areas, the 10 questions in the CHAT tool (64) to explore the patient’s health literacy level, and ACT principles in their communication to focus on the participant’s values and behaviors. The support focus on patients’ central role in managing their disease, empowering them to manage challenges in everyday life. This involves guiding patients to recognize where and when to seek support, monitoring emotional reactions, providing emotional support, reflecting on past success, offering verbal persuasion about capability, feedback on behavior, discussions and reframing in relation to beliefs, fears, avoidance, identity and more. At each face-to-face meeting with the nurse, the defined activities (identified using the PSFS) are evaluated. The nurse can also provide support by telephone or online. At each contact, the mode and time for the next contact are planned together with the participant. The nurses have a chart, developed by the PTs and OTs in the study, describing when it is relevant to consider signposting to a PT or OT. If relevant and the participant is interested, the nurse describes the functional limitations based on the assessment. The support can be face-to-face or by telephone. The nurse can also signpost to a social worker for face-to-face or online support regarding social support opportunities. The nurse coordinates support from other professionals at the hospital and/or in the municipalities to achieve the goals. This include helping to identify relevant existing services within the participant’s own municipality. There is an opportunity for the nurse to arrange a team conference twice during the 6 months with relevant parties from the hospital and/or the patient’s municipality if needed to support goal achievement.
**Close-out consultation**	To evaluate and plan the next steps for achieving future goals	After approximately 6 months, the nurse holds a final consultation with the patient to evaluate the defined activities, achievement of the goals, and how the patient experienced their participation and the possible impact of the intervention. Further needs for support and where are discussed.

The number of hours allocated per participant represents the maximum allowed number to be used during the six-months intervention. PT = physiotherapist; OT = occupational therapist

### A subsequent feasibility study

The developed INSELMA intervention is currently being tested in a feasibility study in accordance with the description of complex interventions [39, 40].

#### Target population

Adults aged 18 years or above diagnosed with RA, PsA, or axSpA for at least 2 years are eligible, to allow the participants to have reached optimal pharmacological treatment. As the intervention targets people with IA experiencing substantial impacts from their disease, we defined that they must answer “no” to Patient Acceptable Symptom State [77–79] and/or report 40 or above on at least two Visual Analogue Scales (0–100) for fatigue, pain, and global assessment of impact of the disease [80]. Moreover, they must have no planned change to disease-modifying anti-inflammatory drugs, planned rehabilitation, ongoing application for early retirement, or planned surgery requiring admission. This is because we consider that these criteria can potentially affect the outcomes of the intervention. In addition, they must not have any unstable psychiatric illness or cognitive impairment, to ensure full and active participation in the intervention. The results from the feasibility study will be reported elsewhere.

### Competence development

The HPs who are to deliver the INSELMA intervention in the subsequent feasibility study are all experienced in rheumatology and are dedicated to delivering the intervention; however, they required some additional training before the initiation of the feasibility study, in accordance with the input from the workshops and the logic model (Fig. 1). Therefore, we planned and conducted a 2-day competence development program for the nurses, and the involved OTs and PTs participated in the first day. The participants received literature about self-management, the management of sleep problems and pain, a short video podcast about the management of fatigue, and the developed tools to use in the intervention ahead of the training. The training was delivered by two experienced psychologists, a social worker and research nurses who are specialists in self-management, self-efficacy, biopsychosocial pain management, health literacy and healthy lifestyle.

The content on the first day encompassed talks and discussions regarding self-management, self-efficacy, opportunities for support from the social system, lifestyle and comorbidities, acknowledgment through communication, anxiety and depression, and an introduction to ACT. The content on the second day encompassed talks about health literacy, management of pain, and talks, group discussions, and practice in ACT principles and acknowledgment through communication.

## Discussion

The objective of this study was to develop an interdisciplinary nurse-coordinated complex self-management intervention as an add-on to usual outpatient care for patients with IA facing substantial impacts. The overarching focus in the intervention was to support these patients’ self-management ability and reduce the impact of their arthritis. The content of the intervention is in accordance with the EULAR recommendations for the implementation of self-management strategies in patients with IA [28]. The recommendations concern encouraging patients to become active partners of the team, patient education, problem solving, goal setting, elements of cognitive behavioural therapy (e.g., ACT), and lifestyle advice to promote physical activity, emotional well-being, work ability, and signpost to relevant support [28].

The scoping review revealed that self-management support can be offered in groups, which is in accordance with role modeling in self-efficacy theory [46]. As we plan to enroll 10 patients from each hospital in the feasibility study, and the recruitment period is expected to be approximately six months, we considered it impossible to deliver group support as part of the intervention. We can consider group-based self-management support when we adjust the intervention and the program theory, based on the results from the feasibility study, before further testing the adjusted intervention.

As part of developing complex interventions, economic considerations are critical, as is identifying key uncertainties or critical points to consider [40]. In Denmark and other European countries, patients with IA do not always have access to a rheumatology nurse and many hospitals cannot offer access to a specialized PT or OT. This could either be because PTs and OTs are not available or because there is no economic coverage to include support from a multidisciplinary team. The INSELMA intervention involves elements of ACT with supervision a couple of times by a psychologist, but access to a psychologist would probably be a superior– but not a feasible– solution. Thus, if the intervention is to become feasible in a wider context, changes are required at the hospital management and political levels.

In the upcoming feasibility study, we aim to address several key uncertainties. These include determining whether the participants perceive the intervention to be meaningful and feasible, understanding how the inter-professional collaboration will work, assessing whether the estimated number of support hours aligns with participants’ needs, and evaluating whether the HPs assigned to deliver the intervention feel adequately qualified to carry out their new roles. The responsibility of conducting a comprehensive biopsychosocial assessment and defining goals and activities together with the participants is new for the nurses. Additionally, working with concepts such as ACT and health literacy is novel for the HPs, who are more familiar with the concepts self-management and self-efficacy. Both the participants in the feasibility study and the HPs who deliver the intervention will be interviewed to explore feasibility, acceptance, fidelity, and resource use. In addition, we will explore whether there are any indications of changes in outcomes in accordance with the program theory. The interviews will explore barriers to and facilitators of the delivery of the intervention and modes of impact (e.g., whether it reflects the logic model), and identify key uncertainties encompassing the identification of participants, logistics, contextual factors, the need for adjustments, and any potential need for additional competence development. The results will reveal whether any of the selected outcome measures indicate the positive changes expected, in accordance with the logic model. The results of the feasibility testing, including evaluation of whether the developed intervention is considered feasible by the HPs and participants, will be reported elsewhere. The results from the feasibility study will be discussed with the local hospital management teams, the HPs who deliver the intervention and the project team, to ensure support to proceed with further testing and implementation. Depending on the results of the feasibility study, the intervention and the program theory will be adjusted before further testing in a larger study.

### Strengths and limitations

The development of the INSELMA intervention in accordance with the MRC framework facilitated close collaboration between the researchers, patient representatives, HPs, and other relevant stakeholders. The process ensured an intervention that is adapted to the local context. We found the framework very useful developing and testing new complex multimodal interventions involving many HPs with various professional backgrounds and from different settings. We could have positioned the intervention in relation to the Chronic Care model [81], but the elements (self-management support, delivery systems design, decision support and clinical information systems, an informed activated patient and a prepared proactive practice team) were also considered during the development process, and are part of the final INSELMA intervention. We believe these elements can lead to improved outcomes for the participants.

Unfortunately, we were unable to combine patient representatives and professionals from the municipalities and hospitals in the same workshops, because of the COVID-19 pandemic. It would have made us able to discuss various perspectives within the same workshops which would have been a significant strength.

Both the two PRPs and the extra patient representatives who participated in the workshops have RA, long disease duration and are in their 50’s and 60’s. Although patient involvement can never be representative of the population, it would be preferable that the patients involved represented a variety of diagnoses and age groups. However, the intervention focused on support to reduce disease impact rather than focusing on the diagnosis, and a scoping review of the literature also informed the development of the intervention.

### Electronic supplementary material

Below is the link to the electronic supplementary material.

Supplementary Material 1

## Data Availability

Data supporting the conclusions presented in this article have been reported in the article. The details of the scoping review are published elsewhere [42, 82].

## List of abbreviations

ACT: Acceptance and commitment therapy
axSpA: Axial spondylarthritis
CBT: Cognitive behavioral therapy
CHAT: Conversational Health Literacy Assessment Tool
EULAR: European Alliance of Associations for Rheumatology
HP: Health professional
IA: Inflammatory arthritis
INSELMA: Interdisciplinary Nurse-coordinated SELf-MAnagement
MRC: Medical Research Council
OSF registries: Open Registries Network
OT: Occupational therapist
PRP: Patient research partner
PSFS: Patient-Specific Functional Scale
PT: Physiotherapist
RA: Rheumatoid arthritis
PsA: Psoriatic arthritis
