# Supplementary material for: Development of a complex Interdisciplinary Nurse-coordinated SELf-MAnagement (INSELMA) intervention for patients with inflammatory arthritis
Source: BMC Health Serv Res. 2024 Jan 17;24:87. doi: 10.1186/s12913-023-10463-1 (PMC10792835; doi:10.1186/s12913-023-10463-1)
Supplement: Supplementary file 1 — Supplementary Material 1 [file 12913_2023_10463_MOESM1_ESM.docx]

**Supplementary Table 1**

Output and outcome measures to be used in the subsequent feasibility study

| **Output** | **Outcome measure** |
| --- | --- |
| Physical disability | Multidimensional Health Assessments Questionnaire (MD-HAQ) (65) |
| Physical activity | Questions from regional questionnaire “How are you?” (characteristics and number of days physically active for 30 minutes or longer) (66) |
| Lifestyle | Smoking habits (never, previous, occasionally, present, and number) and alcohol use (How often do you drink? How often do you drink five units or more on the same occasion?) (67) |
| Impact of the disease | VAS-global assessment of impact of the disease (67)  Patient Acceptable Symptom State (PASS) (77, 79) |
| Pain | VAS-Pain (80) |
| Fatigue | VAS-Fatigue (68) |
| Fatigue coping, severity, and impact | British Rheumatoid Arthritis Fatigue version 2 – numerical rating scales (BRAFv2-NRS) (coping, severity, and impact) (69) |
| Self-efficacy for managing one’s chronic condition and pain | Self-Efficacy for Managing Chronic Diseases – Six-item scale (SEMCD-6IS)  Pain Self-Efficacy Questionnaire (PSEQ) (70) |
| Mental well-being | WHO-5 Well-Being Index (71)  Hospital Anxiety and Depression Scale (HADS) (72) |
| Health-related quality of life | European Quality of Life – five dimensions – five levels (EQ-5D-5L) (73) |
| Health literacy | Health Literacy Questionnaire (HLQ) (74)  Subscale 3 (Actively managing my health; questions 6, 9, 13, 18, and 21) and subscale 6 (ability to actively engage with healthcare providers; questions 2, 4, 7, 15, and 20) |
| Work ability (presenteeism and absenteeism) | Work Productivity and Activity Impairment – General Health (WPAI-GH) (75, 76))  Sickness absence in the past 3 months |

VAS = Visual Analogue Scale
